# Dramatic Efficacy of Interferon and Vemurafenib on Psychiatric Symptoms Revealing *BRAF^V600E^*-Mutated Erdheim–Chester Disease: A Case Report

**DOI:** 10.3389/fimmu.2022.918613

**Published:** 2022-07-06

**Authors:** Jérôme Razanamahery, Maroua Abdallahoui, Guillaume Chabridon, Agnès Fromont, Georges Tarris, Ahmed Idbaih, Pierre Olivier Comby, Francois Godard, Julien Haroche, Sylvain Audia, Bernard Bonnotte

**Affiliations:** ^1^ Department of Internal Medicine and Clinical Immunology, Dijon University Hospital, Dijon, France; ^2^ Department of Psychiatry, Dijon University Hospital, Dijon, France; ^3^ Department of Neurology, Dijon University Hospital, Dijon, France; ^4^ Department of Pathology, Dijon University Hospital, Dijon, France; ^5^ Sorbonne Université, Institut du Cerveau - Paris Brain Institute - ICM, Inserm, CNRS, AP-HP, Hôpital Universitaire La Pitié Salpêtrière, DMU Neurosciences, Paris, France; ^6^ Department of Neuroradiology, Dijon University Hospital, Dijon, France; ^7^ Nuclear Medicine Department, Centre Georges Francois Leclerc, Dijon, France; ^8^ Department of Internal Medicine 2, National Reference Center for Histiocytosis, Pitié-Salpétrière Hospital, Paris, France

**Keywords:** histiocytosis, Erdheim-Chester disease, psychosis, cerebellar syndrome, interferon, BRAF inhibitor

## Abstract

Erdheim–Chester disease (ECD) is a rare condition with underestimated neurological involvement. Mild psychiatric symptoms such as mood swings have been rarely described in the clinical spectrum of neuro-ECD. We here describe the first patient with psychiatric manifestations of delirium revealing ECD with neurological involvement with favorable evolution under interferon followed by BRAF inhibitor monotherapy. An 81-year-old woman was referred to the hospital because of delirium and severe cognitive impairment associated with a cerebellar syndrome. Brain magnetic resonance imaging showed “FLAIR-changes” lesions in the pons and upper cerebellum peduncles. Blood and cerebrospinal fluid (CSF) analyses showed normal results except for an elevated neopterin level in the CSF. Whole-body CT scan (^18^FDG-PET) showed peri-nephric fat infiltration and aorta adventitia sheathing with radiotracer uptake in the pons, vessels, peri-nephric fat, and bone lesions, which was characteristic of ECD. The diagnosis was confirmed on perirenal tissue biopsy, which also showed a *BRAF^V600E^* mutation. Treatment with interferon resulted in the resolution of delirium, and treatment with BRAF inhibitor subsequently resulted in a partial remission of all active sites. This case highlights that delirium can be the first manifestation of neurodegenerative ECD. ECD should be screened in unexplained psychiatric features as interferon and targeted therapy appear to be effective in this situation.

## Introduction

Erdheim–Chester disease (ECD) is a rare clonal histiocytosis characterized by clinical and radiological features (long bone and sinusal osteosclerosis, peri-nephric fat infiltration, cardiovascular involvement) associated with a compatible histology (1)(CD68+, CD1a- tissue infiltrate with frequent mutation of the mitogen-activating protein kinase pathway gene). Neurological involvement is underestimated and mainly represented by isolated or combined “mass-like” or vascular lesions (2). Patients may also suffer from associated neurodegeneration (3). The diagnosis of neuro-ECD is based on magnetic resonance imaging showing most often “FLAIR-signal changes” in meninges, throughout the cerebrum and cerebellum, or the brain parenchyma of the hypothalamic–pituitary axis (2). The neurological signs are protean and consist mainly of a cerebellar disability, cognitive impairment, and/or pyramidal tract symptoms depending on the location and mechanism of the lesions. However, psychiatric manifestations (i.e., delirium, hallucinations) are exceptional in the spectrum of neuro-ECD, mainly reported as mood disorders (4–6). Despite emerging targeted therapies, neuro-ECD’s prognosis remains poor (7), particularly in patients suffering from neurodegenerative forms.

We describe the first case of neuro-ECD with delirium and psychiatric symptoms as initial symptom regressing with ECD-specific therapies.

## Methods

This case report did not require an institutional review board according to the French legislation and was conducted in accordance with the declaration of Helsinki.

Histology was performed on 4-µm-thick tissue sections after staining with hematoxylin and eosin and immunohistochemistry, including at least CD1a, S100, and CD68 primary antibodies. Detection of mutations was performed on tissue biopsies infiltrated by histiocytosis. Tumor DNA was extracted from formalin-fixed and paraffin-embedded tissues. Detection of *BRAF^V600E^* mutation was performed using picodroplet digital PCR (8).

The search for paraneoplastic syndromes was performed using a panel of antibodies to *GAD-65*, *Zic-4*, *Tr*, *SOX1*, *Ma1*, *Ma2*, *CV2*, *Ri*, *Yo*, *CV-2*, and *HuD*. The panel was tested on blood and CSF samples.

## Clinical Case

An 81-year-old woman was referred to the hospital for delirium, severe cognitive impairment, and cerebellar syndrome. She had a medical history of central retina vein occlusion and Grave’s disease and was not taking any medication. The neuropsychiatric problem began 8 months earlier with delirium combining auditory and visual hallucinations requiring hospitalization. At that time, the neurological examination showed a static and dynamic cerebellar syndrome. Cerebral computed tomography (CT) scan and electroencephalogram showed unremarkable results. Analysis of blood samples showed normal results. The patient was considered having an age-related cognitive decline and was discharged with antipsychotic treatments (olanzapine). Delirium and neurological disability remained stable over time. The worsening of delirium with persistent delusional persecution syndrome motivated a new hospitalization. The neurological examination showed a cerebellar syndrome with unstable ataxic gait, stance disturbance, abnormal finger chase, and nose–finger chase without speech disturbance. The Scale for Assessment and Rating of Ataxia (SARA) score was 14. The patient also had pseudobulbar features. She had no associated tremor/asterixis or extrapyramidal or pyramidal features. She had no loss of strength or sensitivity and no cranial nerve palsies. Psychiatric features included a delirium with delusional persecution syndrome with auditive and visual hallucinations not modified by circadian rhythm. The patient had complete anosognosia and mild cognitive impairment (Mini-Mental State Examination: 22/30). Extra-neurological features included mild dyspnea (class II New York Heart Association classification) secondary to pericardial effusion.

**Figure 1:**
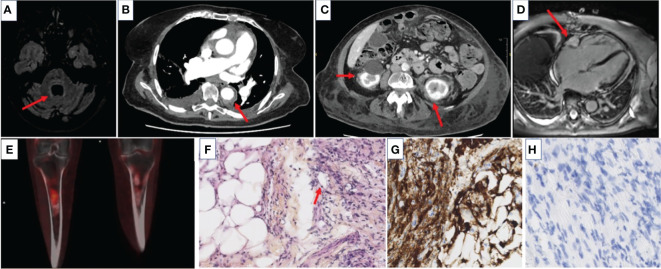
Radiological and histological features of the patient suffering from Erdheim–Chester disease (ECD) at diagnosis. **(A)** Axial brain MRI showing hyperintense signal in the pons on T2-FLAIR sequence. **(B)** Axial CT scan showing sheathing of aorta adventitia. **(C)** Axial CT scan showing bilateral perinephric fat infiltration defined as “hairy-kidney”. **(D)** Axial heart MRI showing a right atrium mass suggestive of a pseudo-tumor. **(E)** Sagittal ^18^F fluorodeoxyglucose positron emission tomography demonstrating radiotracer uptake in meta-diaphysis. **(F)** Histopathological analysis of a CT scan-guided peri-renal adipose tissue biopsy showing massive infiltration by numerous foamy histiocytes and small lymphocytes, with features of cyto-steatonecrosis (HES, magnification ×300). **(G)** Similar biopsy sample with immunostaining showing CD163-positive histiocytes (brown staining, magnification ×300). **(H)** Sample biopsy showing the absence of CD1a-positive cells (brown staining, magnification ×300).

**Figure 2:**
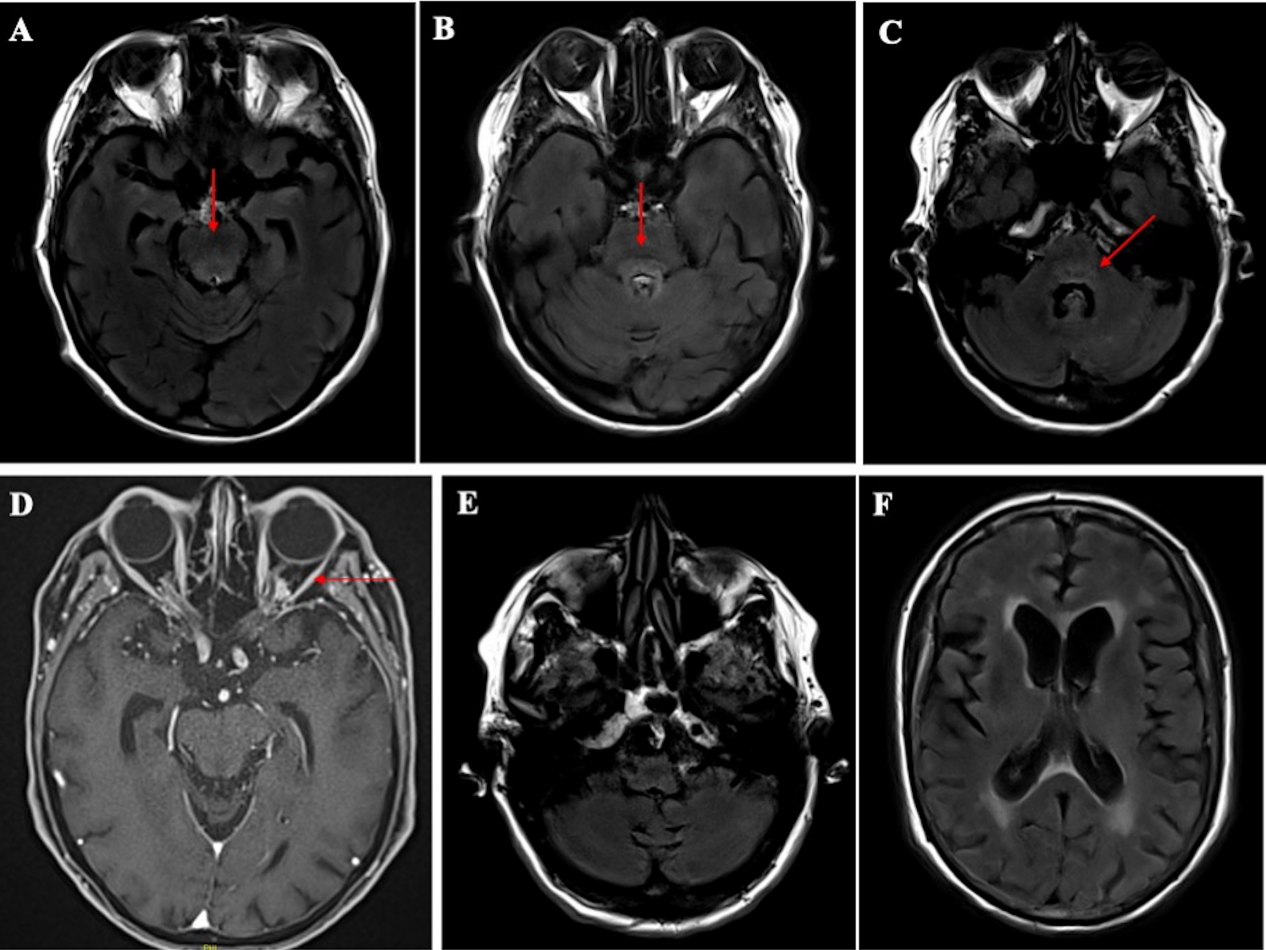
Radiological brain imaging of the ECD patient at diagnosis. **(A)** Axial brain MRI showing a hyperintense signal in the pons on the T2-FLAIR sequence. **(B)** Axial brain MRI showing a hyperintense signal in the pons on the T2-FLAIR sequence. **(C)** Axial brain MRI showing a slight hyperintense signal on the T2-FLAIR sequence on the upper cerebellum. **(D)** Axial brain MRI showing left orbital pseudotumor in fat-saturated post-gadolinium T1 imaging. **(E)** Axial brain MRI showing no signs suggestive of cerebellum atrophy on T2 FLAIR sequence. **(F)** Axial brain MRI diffuse cortical atrophy predominant in the parietal lobes, with leukoaraiosis (Fazekas grade II) of presumed vascular origin.

Because of the persistent delirium and cerebellar syndrome, brain magnetic resonance imaging (MRI) was performed. It showed “FLAIR-signal changes” in the pons and upper cerebellum peduncles and a left orbital pseudotumor (**Figures 1**, **2**). The patient also underwent repeated electroencephalograms showing no seizures. Blood tests for infections (HIV, HBV, HCV), immune-mediated inflammatory diseases (antinuclear antibodies, ANCA, cryoglobulinemia, angiotensin-converting enzyme), vitamin deficiencies (B1/B6/B9/B12 vitamins), or drug or toxic intake showed unremarkable results. Renal, hepatic, and thyroid functions were normal, and there were no metabolic abnormalities (calcium, magnesium). CSF analysis showed one leukocyte/mm (3), seven red blood cells/mm (3), and a protein level of 0.35 g/l. Search for oligoclonal bands and antibodies associated with paraneoplastic syndrome showed negative results. The tau protein level was 274 ng/l (N < 400), and the phosphorylated isoform level was 33 ng/l (N <60). The neopterin level was 9.4 nmol/l (N <5).

A whole-body CT scan was performed to rule out cancer and showed bilateral peri-nephric fat infiltration and aortic vascular sheathing (**Figure 1**). The ^18^FDG-PET scan revealed radiotracer uptake in the pons, right atrium, aorta adventitia, and peri-nephric fat. It also showed bilateral symmetric osteosclerosis of long bones, highly suggestive of ECD. Perinephric fat biopsy confirmed the diagnosis of ECD with tissue infiltration by fibrosis and foamy CD68^+^ CD1a^-^S100^-^ histiocytes. Pyrosequencing revealed a *BRAF^V600E^* mutation.

The patient first received only interferon-alpha (180 µg/weekly for 2 weeks) at the beginning, allowing a rapid regression of the hallucinations and the malignant state after 10 days. After molecular status establishment, the patient received a *BRAF* inhibitor (vemurafenib 420 mg twice daily) without the MEK inhibitor for aggressive multisystemic ECD.

**Figure 3:**
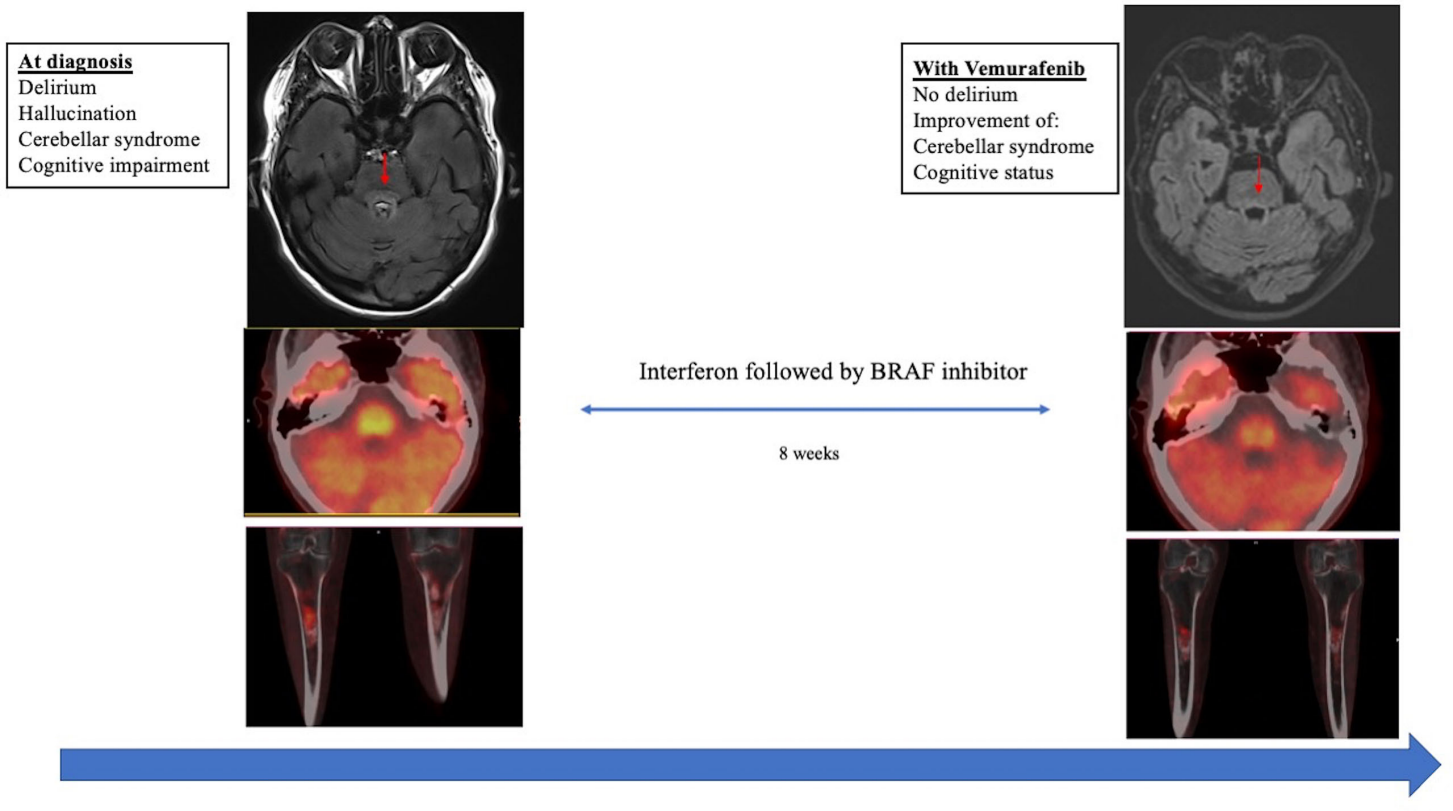
Radiological and metabolic evolution of the ECD patient before and after vemurafenib. On the left side of the figure: imaging at ECD diagnosis before specific treatment. From top to bottom: axial brain MRI showing FLAIR change lesions in the pons at ECD diagnosis (upper image). Axial ^18^F fluorodeoxyglucose positron emission tomography at diagnosis showing radiotracers in the pons (middle image) and in long bones at ECD diagnosis (bottom image). On the right side of the figure: imaging after 8 weeks treatment with vemurafenib. From top to bottom: axial brain MRI showing a decrease in the intensity of the FLAIR signal in the pons after vemurafenib (upper image). Axial ^18^F fluorodeoxyglucose positron emission tomography after vemurafenib showing a slight decrease of radiotracer uptake in the pons (middle image) and in long bones (bottom image) consistent with partial metabolic response using PERCIST criteria.

At 8 weeks of follow-up, neurological examination showed complete regression of delirium with a slight improvement in cerebellar syndrome on gaiting (SARA score: 12) and cognitive status (MMSE: 26/30) (**Table 1**). Brain MRI showed partial regression of lesions in the pons. ^18^FDG-PET showed a decrease in radiotracer uptake in all ECD sites consistent with a partial metabolic response according to PERCIST criteria (**Figure 3**).

**Table 1:** Evolution of the neuropsychiatric tests at diagnosis and after vemurafenib.

Neuropsychiatric test	ECD diagnosis	After vemurafenib
MMSE	22/30	26/30
SARA score	14	12
FAB	14/18	16/18
The Clock-Drawing Test	3/30	20/30
The 5-word test	19/20	20/20

MMSE, Mini-Mental State Examination; SARA, Scale for Assessment and Rating of Ataxia; FAB, Frontal Assessment Battery.

## Discussion

To our best knowledge, we report the first presentation of neuro-ECD with delirium including visual and auditive hallucinations as the main initial symptom.

The neurological spectrum of ECD mainly involves the hypothalamic–pituitary axis, the cerebrum/cerebellum, the meninges, or the brain parenchyma, leading to endocrinopathies and/or neurological deficit (2). Manifestations are heterogeneous, ranging from fatigue to stroke, mainly depending on ECD locations and presentation. Pachymeningeal thickening may cause diffuse plaque extension in the dural structure, mimic meningioma, or involve the spinal cord (9). “Mass-like “lesions mainly affect all structures of CNS. Degenerative lesions may be responsible for “FLAIR-signal” changes within the posterior fossa (i.e., corpus medullare, dentate nuclei) and atrophic changes in the cerebellum or the brain stream (2, 4, 5). Notably, intermittent neurologic manifestations such as uncontrolled crying, laughing, or more generally inappropriate behavior related to pseudobulbar palsy have been reported in a cohort of ECD patients, but none of them had pure psychiatric symptoms related to ECD (10). Mood swings have also been previously described and may be secondary to interferon therapy or ECD-related fatigue (10). Mild cognitive impairment has been reported in patients with ECD, and a few patients have progressed to dementia (6). Cognitive impairment is also described in other histiocytic neoplasms. It is mainly reported in Langerhans-cell histiocytosis (11), another histiocytosis with a frequent *BRAF^V600E^* mutation. It is rarely described in Rosai–Dorfman disease (12), another histiocytosis with rarer clonal involvement (13). These histiocytoses affect younger people, mostly young adults (even children) compared with ECD. The mechanism of cognitive decline is still unclear in histiocytic neoplasms but may be related to *BRAF^V600E^*-induced microglia assault *in utero* (3, 14) (at least in ECD and LCH).

In our clinical case, the onset of psychiatric disorders was suggestive of an organic disease because of age, cerebellar involvement, and resistance to antipsychotic treatment. In our patient, the cerebellar disability was crucial for the diagnosis, since the acquired conditions affecting the cerebellum at that age are restricted (15), including drugs and toxics; inflammatory, vascular, and metabolic diseases; infections; and neoplastic diseases, among which are histiocytoses.

The diagnosis of neuro-ECD is based on the MRI presentation, the exclusion of differential diagnoses (i.e., paraneoplastic syndrome, infections, vasculitis, metabolic disturbance), and normal CSF analysis (2) in patients with proven ECD. Meanwhile, the neopterin level was elevated in our presentation. The elevation of neopterin in the CSF has been reported in several disorders, including acute viral and bacterial infections and chronic neuroinflammatory diseases (16, 17) but not dementia (18). Neopterin is a neurological biomarker of brain inflammation mainly produced by activated macrophages and microglia after interferon-gamma exposure (19). CSF neopterin is also elevated in patients with hemophagocytic lymphohistiocytosis (20). In this case, the elevation of neopterin in CSF could directly reflect histiocytic activation during histiocytosis since the cultures were sterile and other neurologic conditions were ruled out.

In this presentation, the pons lesion on brain MRI is responsible for the cerebellar syndrome and the pseudobulbar features but does not explain the delirium. In literature, high-resolution functional brain MRI in ECD patients shows a diffuse reduction of cortical thickness and gray matter (21) as described in patients with a high risk of psychosis (22). In this case, we hypothesize that psychotic presentation might be related to a progressive diffuse reduction of cortical thickness related to ECD. In this case, the initial efficacy of interferon on delirium is a strong argument for the causality of histiocytosis in psychiatric symptoms.

Treatment of ECD, which depends on staging and molecular status (1), is based on conventional agents (pegylated interferon, mammalian target of rapamycin inhibitors), biological agents (interleukin-1 or tumor necrosis factor alpha inhibitors), or targeted therapies (BRAF/MEK inhibitors) in life-threatening situations (1).

Targeted therapies (BRAF or MEK inhibitors) are recommended for neurological forms of histiocytosis, although drug delivery to the CNS is incomplete, making therapeutic response unpredictable and heterogeneous. Furthermore, imaging improvement does not always correlate with clinical response, especially in neurodegenerative forms. Because discontinuation of targeted therapy is associated with relapse (23), *BRAF* inhibitor treatment should be continued with a possible dose reduction after sustained metabolic remission.

The main interest of this clinical case presentation is the observation of delirium revealing a multisystemic ECD with a favorable outcome with specific therapies. This will improve the panel of differential diagnoses in the setting of unexplained encephalopathy. However, it should be noted that this is only one case report and our results have to be enriched by other reports of delirium in ECD patients including the outcomes with targeted therapy treatments. We also regret the absence of high-resolution functional brain imaging to evaluate cortical thickness change with treatment to corroborate our hypothesis on the nature of the psychosis.

However, this case report highlights that ECD should be screened in patients with unexplained delirium, as treatment with interferon followed by BRAF inhibitor improves psychiatric features and induces partial remission in neurodegenerative ECD.

## Conclusion

This report details the first case of delirium as initial manifestation of neurodegenerative ECD resolutive with ECD-specific therapies.

## Data Availability Statement

The original contributions presented in the study are included in the article/supplementary material. Further inquiries can be directed to the corresponding author.

## Ethics Statement

The studies involving human participants were reviewed and approved by Dijon University Hospital Ethics Committee. The patients/participants provided their written informed consent to participate in this study. Written informed consent was obtained from the individual(s) for the publication of any potentially identifiable images or data included in this article.

## Author Contributions

JR and MA wrote the initial draft. All authors provided primary care for the patient, critically reviewed the manuscript for the scientific content, and approved the final version of the manuscript.

## Conflict of Interest

The authors declare that the research was conducted in the absence of any commercial or financial relationships that could be construed as a potential conflict of interest.

## Publisher’s Note

All claims expressed in this article are solely those of the authors and do not necessarily represent those of their affiliated organizations, or those of the publisher, the editors and the reviewers. Any product that may be evaluated in this article, or claim that may be made by its manufacturer, is not guaranteed or endorsed by the publisher.
